# Association between Cigarette Smoking Frequency and Health Factors among Korean Adults

**Published:** 2018-07

**Authors:** Hu-Nyun KIM, Mi-Ae SHIN, Jae-Hun ROH, Mi-Kyung HAN, Yu-Mi WON, Ik-Rae CHO, Hyo-Joo PARK, Taek-Kyun LEE, Tae-Keun PARK, Hee-Moon HA, Seung-Won YANG, Seung-Hi MIN, Shin-Young LEE, Sang-Ho LEE, Ji-Hyuk KIM, Se-Jeong KWON, Yeon-Sook LEE, Young-Wan KO, In-Hong KIM, Jeong-Hyeon KWAK, Tae-Gyeom JUNG, Jeong-Woo JEON, Kyung-Rok OH, Hye-Sook HA, Me-Suk KIM, Yeong-Man KIM, Min-Jeong KIM, Tae-Young KIM, Ji-Hyoung CHIN

**Affiliations:** 1. College of Creative Future Talent, Daejin University, Gyeonggi-do, Republic of Korea; 2. College of Education, Hankuk University of Foreign Studies, Seoul, Republic of Korea; 3. Dept. of Sports and Leisure, Kwangju University, Gwangju, Republic of Korea; 4. College of Sport Sciences, Chung-Ang University, Gyeonggi-do, Republic of Korea; 5. Dept. of Exercise & Rehabilitation, Daewon University, Chungcheongbuk-do, Republic of Korea; 6. Dept. of Social Physical Science, Chodang University, Jeollanam-do, Republic of Korea; 7. College of Medicine, Dongguk University, Gyeongsangbuk-do, Republic of Korea; 8. Dept. of Taekwondo, Gachon University, Gyeonggi-do, Republic of Korea; 9. College of Physical Education, Kyunghee University, Gyeonggi-do, Republic of Korea; 10. College of Convergence Culture and Arts, Sungshin Women’s University, Seoul, Republic of Korea; 11. Division of Sports, SoongSil University, Seoul, Republic of Korea

**Keywords:** Smoking frequency, Health factor, Korea

## Abstract

**Background:**

Recently, there has been a trend that cigarette smoking rate in Asian and Africa adults has increased while the age group to start smoking has decreased gradually. This study aimed to investigate the relationships between lifetime smoking and hypertension, diabetes, obesity, waist measure, fasting blood pressure and food consumption, in order to look into health status depending on smoking status in Koreans.

**Methods:**

Totally, 1075 men and 697 women with no disease participated in this study, in which one-way ANOVA was conducted by using SPSS version 18.0 for statistical process. The level of statistical significance was 0.05.

**Results:**

As a result of analysis on relationship between lifetime smoking and hypertension, obesity and diabetes, statistically significant differences were revealed.

Lifetime smoking was found to be significantly associated with increased waist measure, higher level of fasting blood sugar, and more ingestion of nutrients (carbohydrate, fat, and protein).

**Conclusion:**

Increased amount of lifetime cigarette smoking was shown to negatively influence various health factors, which might become to be a drive to cause diseases. Therefore, method to improve health factors must be sought for via education and campaign to control an amount of cigarette smoking in Korean adults.

## Introduction

Cigarette smoking is known as the cause of death at the highest rate worldwide (1), which is reported by WHO to lead to 5,000,000 deaths by 2008 and 8,000,000 deaths by 2030. One, per 6 seconds, dies of cigarette smoking (2). It is an important factor to cause various diseases including cardiovascular disease. Naturally every country permits cigarette with limitation posed to the adolescence, while advertisements regarding a number of types of diseases caused by cigarette smoking are commonly seen. Even though cigarette is sold in a number of routes according to the national environment and culture, smoking rate has been decreasing in countries with higher income (3, 4). However, it has been increasing in countries with lower income (Asia, Africa) (5).

Smoking has been diversified by age, gender and occupation in South Korea and smoking population has not been decreasing in reality. In order to overcome this situation, studies under various categories and continual anti-smoking campaign should be conducted. In addition, as the number of studies on health factors recently has increased, effect of smoking and drinking behavior on income status of men and women (6), relationship between an amount of cigarette smoking and mental health (7) and smoking problems in adolescence (8–11) are mainly under study in south Korea. However, data regarding the effect of cigarette smoking on health factors in Korean adults are insufficient. Accordingly, it would be significant to compare and observe overseas studies on cigarette smoking and health factors as well as to report current health status of Korean adults by looking into effect of lifetime smoking on hypertension, obesity, diabetes, waist measure, fasting blood sugar and ingestion of nutrients (carbohydrate, lipid, protein), in terms of data provision for public health education. Therefore, it needs to alert domestic smokers to risk of smoking by looking into relationship between cigarette smoking and disease. Deferring from Western people whose enjoy meat diet as a routine meal, it would be meaningful to trend of studies on effect of cigarette smoking on health factors in Koreans as the Asian whose routine meal consists of vegetables.

The purpose of this study was to warn the risk of smoking by analyzing the relationship of hypertension, obesity, diabetes and fasting blood sugar according to lifetime smoking in Koreans.

## Methods

### Data collection

This study used data obtained from subjects who participated in the 6th National Nutrition Survey (2013–2015) conducted by Korea Centers for Disease Control and Prevention. The data consists of health questionnaire, examination questionnaire and nutrition questionnaire. Health and examination questionnaires were conducted at the mobile examination center, while nutrition questionnaire was conducted via personal interview as the nutrition research team visited subject households in person. The research items of this study were gender, age, smoking status, weight, waist measure and fasting blood glucose of the subjects. This study was based on an approval obtained from Korea National Health and Nutrition Examination Survey (Project No. 2013-12EXP-03-5C).

### Study subjects

Total 1,772 subjects consisting of 1,075 men and 697 women participated in this study (Table 1).

**Table 1:** General characteristics of study subjects by lifetime smoking

***Variable***	***N (%)***	***Less than 100 cigarettes***	***100 cigarettes or more***	***Non-smoking***	***χ^2^***
Gender					629.770***
Men	697	33	481	183	
Women	1075	31	133	911	
Sum	64	614	1094	1772	
Age(yr)					29.506***
20s	370	21	97	252	
30s	672	27	259	386	
40s	664	16	242	406	
50s	66	0	16	50	
Sum	1772	64	614	1094	

*P*<.001***

### Statistical analysis

Data obtained from this study was analyzed using SPSS for windows version 18.0 (Chicago, IL, USA) as the following. General characteristics corresponded to types of smoking were presented by descriptive statistics. Effect of lifetime smoking on disease risk factors such as hypertension, obesity, diabetes in Korean adults was characterized by chi-square test. Waist measure and fasting blood glucose by lifetime smoking were analyzed by one-way ANOVA. The level of statistical significance for all analysis was 0.05.

## Results

### Relation to hypertension, diabetes and obesity by lifetime smoking

Cross-analysis was conducted to determine difference in hypertension, obesity and diabetes per lifetime smoking (Table 2). As a result of analysis on relationship between lifetime smoking and hypertension, obesity and diabetes, statistically significant differences were revealed.

**Table 2:** Result of cross-analysis on hypertension, diabetes and obesity per lifetime smoking

***Variable***	***N (%)***	***Less than 100 cigarettes***	***100 cigarettes or more***	***non-smoking***	***χ^2^***
Status of hypertension					83.881
Normal	1188(100)	42(3.5)	330(27.8)	816(68.7)	***
Stage towards hypertension	396(100)	16(4.0)	175(44.2)	205(51.8)	
Hypertension	184(100)	6(3.3)	105(57.1)	73(39.7)	
Sum	1768(100)	64(3.6)	610(34.5)	1094(61.9)	
Status of obesity					47.195
Low weight	108(100)	5(4.6)	28(25.9)	75(69.4)	***
Normal	1181(100)	45(3.8)	358(30.3)	778(65.9)	
Obesity	483(100)	14(2.9)	228(47.2)	241(49.9)	
Sum	1772(100)	64(3.6)	614(34.7)	1094(61.7)	
Status of diabetes					44.376
Normal	1417(100)	53(3.7)	439(31)	925(65.3)	***
Stage towards diabetes	294(100)	9(3.1)	140(47.6)	145(49.3)	
Diabetes	61(100)	2(3.3)	35(57.4)	24(39.3)	
Sum	1772(100)	64(3.6)	614(34.7)	1094(61.7)	

*P*<.001***

### Analysis on relationship between lifetime smoking and waist measure

The result of analysis on group difference by lifetime smoking in Korean adults is provided in the Table 3. Statistically significant difference between groups was shown as a result of the analysis. Waist measure was highest in the 100 cigarettes or more smoking group in lifetime, while in less than 100 cigarettes smoking group and non-smoking group in descending order. Cigarette smoking appeared to increase waist measure as a result of the analysis.

**Table 3:** Result of ANOVA on relationship between lifetime smoking and waist measure

***Variable***	***N***	***M***	***SD***	***F***
*Status of lifetime smoking*				89.119***
Less than 100 cigarettes	64	77.41	10.20	
100 cigarettes or more	614	82.83	9.76	
Non-smoking	1094	76.40	9.43	

*P*<.001***

### Analysis on relationship between lifetime smoking and fasting blood glucose

The result of analysis on group difference by lifetime smoking in Korean adults is provided in the Table 4. Statistically significant difference between groups was shown as a result of the analysis. Fasting blood glucose was shown to be higher in 100 or more cigarette smoking group than non-smoking group.

**Table 4:** Result of ANOVA on relationship between lifetime smoking and fasting blood glucose

***Variable***	***N***	***M***	***SD***	***F***
*Status of lifetime smoking*				19.824***
Less than 100 cigarettes	64	94.22	15.52	
100 cigarettes or more	614	98.55	23.95	
Non-smoking	1094	92.57	15.46	

*P*<.001***

### Relationship between degrees of lifetime smoking and amount of food ingestion

One-way analysis of variance (ANOVA) was carried out to identify the relationship between lifetime smoking and amount of food ingestion. Statistically significant inter-group differences between groups distinguished by the degree of lifetime smoking were found. Results are summarized in Table 5. The group of subjects with lifetime smoking by more than 100 cigarettes per day and the group of lifetime smokers with less than 100 cigarettes a day appeared to have higher levels of food intake than the group of non-smokers. This suggests that increase in the amount of food intake could be attributable to smoking.

**Table 5:** Results of ANOVA between degrees of lifetime smoking and amount of food

***Variable***	***N***	***M***	***SD***	***F***
Degrees of lifetime smoking	64	2333.80	903.51	63.677***
Less than 100 cigarettes	614	2475.64	1082.98	
100 cigarettes or more Non-smoking	1094	1962.25	799.92	

*P*<.001*** // Ingestion

### Relationship between degrees of lifetime smoking and amount of energy intake

One-way ANOVA was carried out to determine the relationship between lifetime smoking and amount of energy intake. Statistically significant inter-group differences between groups distinguished by the degree of lifetime smoking were found. Results are summarized in Table 6. The group of subjects smoked more than 100 cigarettes per day appeared to have higher levels of energy intake as shown in Table 6. Inter-group differences were then examined. The group of lifetime smoking with more than 100 cigarettes per day appeared to have higher levels of energy intake.

**Table 6:** Results of ANOVA between degrees of lifetime smoking and amount of energy intake

***Variable***	***N***	***M***	***SD***	***F***
Degrees of lifetime smoking	64	80.86	37.52	38.738***
Less than 100 cigarettes	614	87.77	47.48	
100 cigarettes or more Non-smoking	1094	70.10	35.30	

*P*<.001***

### Relationship between degrees of lifetime smoking and intake levels of nutrients (protein, fat and carbohydrate)

The relationship between degrees of lifetime smoking and intake level of nutrients (protein, fat, and carbohydrate) was examined. Significant relationships were found. The group of subjects who smoked more than 100 cigarettes a day appeared to have higher levels of all nutrients than the group of non-smokers (Table 7). In terms of ingestion of carbohydrate, a significant intergroup difference was found. The group of subjects smoked less than 100 cigarettes a day appeared to have higher levels of carbohydrate ingestion than the group of non-smokers.

**Table 7:** Results of ANOVA between degrees of lifetime smoking and intake amount of nutrients

***Variable***	***N***	***M***	***SD***	***F***
Protein				38.738
Less than 100 cigarettes	64	80.86	37.52	
100 cigarettes or more	614	87.77	47.48	
Non-smoking	1094	70.10	35.30	
Fat				19.273***
Less than 100 cigarettes	64	54.22	33.67	
100 cigarettes or more	614	61.26	40.37	
Non-smoking	1094	49.65	35.29	
Carbohydrate				21.651***
Less than 100 cigarettes	64	333.78	17.00	
100 cigarettes or more	614	332.43	5.21	
Non-smoking	1094	293.44	3.52	

P<.001***

## Discussion

The purpose of this study is to warn the risk of smoking by analyzing the relationship of hyper-tension, obesity, diabetes and fasting blood sugar according to lifetime smoking in Koreans Occurrence of hypertension was shown to be higher in people smoking 100 or more cigarettes in lifetime than non-smokers, in Korean adult men and women (age 20-60 yr). It suggests relation to blood vessel, for which vascular elasticity is considered as important. Vascular elasticity is not only directly related to hypertension, arteriosclerosis, cerebrovascular disease and cardiovascular disease, but also reported to more decrease due to few change in blood flow rate as arterial pulse wave velocity and physical activities decrease over aging (11). In turn, vascular elasticity is reported to gradually decrease over aging (12) and accelerated by an amount of cigarette smoking, resulting in increased blood pressure. As cigarette smoking generally increases an amount of fat at the core of body (abdomen), i.e., waist measure as well as body fat (% fat), which is remarkable in women rather than in men (13,14). In this study, however, almost no difference in obesity of smokers was found compared to that of non-smoker. It might be because the standard of lifetime smoking was set as 100 cigarettes. Difference in status of obesity would be remarkable if comparing to heavy smoker. In the study regarding effect of smoking and obesity on depression (15), smoking and depression appeared to be closely related each other in office workers at age <40, suggesting that smoking and obesity could be risk factors of depression in workers. Therefore, it implies that enhanced anti-smoking/weight managing program could reduce symptoms of patients with depression in terms of health management. As heavier smokers are known to be highly exposed to risk of diabetes (16), so occurrence of diabetes was shown to be higher in relatively heavy smokers in lifetime compared to non-smokers. Generally if quitting smoking in sudden, risk of diabetes was reported to become high (17) in addition to increase in weight (18, 19). As quitting smoking leads to increase in weight in women rather than in men, risk of exposure to diabetes could become high in women. It is consistent with the result of study suggesting that an amount of abdominal fat tissues, so an amount of fat at abdomen and waist increases (20) as an amount of cigarette smoking increases. Fasting blood glucose was shown to be significantly high in subjects with 100 or more cigarette smoking in lifetime, compare to non-smokers. Long-term glucose concentration control marker is glycosylated hemoglobin A1c (HbA1c). As this HbA1c is used as one of the diabetes factors (21), so it might be important in relation to fasting blood glucose. Therefore, cigarette smoking increases diabetes and insulin resistant risk factors (22, 23), so resulting in high fasting blood glucose level. Similarly, disease factors associated with cigarette smoking are presented as diverse in Asians including Koreans compared to Western people whose main staple food is meat and as different among Asians especially between East and South Asians whose geographic locations are different.

Results of ANOVA revealed that smokers had significant higher levels of food ingestion, energy intake, and nutrients ingestion than nonsmokers. In particular, the ingestion of lipid and carbohydrate appeared to be increasing. This contradicts with results of previous studies (24–26) suggesting suppression of food intake by nicotine substance from smoking. Subjects participated in the present study were lifetime smokers who smoked either more than 100 cigarettes or less than100 cigarettes a day while non-smokers also included those who had quit smoking. Thus, the increase in weight, body fat, and BMI might be attributable to the mechanism of post-smoking dietary control. These results suggest that it is important for adolescents to recognize the importance of non-smoking so that they can secure their healthy bodies free from obesity.

Increased amount of cigarette smoking results in increased body fat or BMI, and increased BMI is closely related to emergence of cardiovascular disease. With increased BMI, mortality due to cardiovascular disease was found to be high in East Asian countries compared to South Asian countries (27), it is because that food intake is mainly meat in South Asians whose digestive enzymes are developed well, compared to East Asians, which demands for multidirectional study. In conclusion, increased amount of lifetime cigarette smoking was shown to negatively influence various health factors (hypertension, obesity, diabetes, waist measure, fasting blood sugar and nutrients ingestion), which might become to be a drive to cause diseases. Therefore, burden from medical expenses should be reduced by improving health factors via anti-smoking education and campaign in Korean adolescence to adults.

Further studies need to look into effect of physiological cerebral mechanism caused by cigarette smoking on health factors. In addition, experiment specifically designed for an amount of smoking in adult men and women is expected to useful to obtain definite results regarding relationship between cigarette smoking and health factors.

## Conclusion

Increased amount of lifetime cigarette smoking was shown to negatively influence various health factors, which might become to be a drive to cause diseases. Therefore, we should strive to recognize the importance of health by continuing systematic preventive education and publicity activities on smoking cessation of young people.

## Ethical considerations

Ethical issues (Including plagiarism, informed consent, misconduct, data fabrication and/or falsification, double publication and/or submission, redundancy, etc.) have been completely observed by the authors.
